# Artichoke (Cynara Scolymus) Methanolic Leaf Extract Alleviates Diethylnitrosamine-Induced Toxicity in BALB/c Mouse Brain: Involvement of Oxidative Stress and Apoptotically Related Klotho/PPARγ Signaling

**DOI:** 10.3390/jpm12122012

**Published:** 2022-12-04

**Authors:** Betul Cicek, Sidika Genc, Yesim Yeni, Mehmet Kuzucu, Ahmet Cetin, Serkan Yildirim, Ismail Bolat, Mecit Kantarci, Ahmet Hacimuftuoglu, Georgios Lazopoulos, Aristidis Tsatsakis, Konstantinos Tsarouhas, Ali Taghizadehghalehjoughi

**Affiliations:** 1 Department of Physiology, Faculty of Medicine, Erzincan Binali Yildirim University, Erzincan 24100, Turkey; 2 Department of Medical Pharmacology, Faculty of Medicine, Bilecik Seyh Edebali University, Bilecik 11230, Turkey; 3 Department of Medical Pharmacology, Faculty of Medicine, Ataturk University, Erzurum 25240, Turkey; 4 Department of Biology, Faculty of Arts and Sciences, Erzincan Binali Yildirim University, Erzincan 24100, Turkey; 5 Department of Biology, Graduate School of Natural and Applied Sciences, Erzincan Binali Yildirim University, Erzincan 24100, Turkey; 6 Department of Pathology, Faculty of Veterinary Medicine, Ataturk University, Erzurum 25240, Turkey; 7 Department of Radiology, Faculty of Medicine, Erzincan Binali Yildirim University, Erzincan 24100, Turkey; 8 Department of Radiology, Faculty of Medicine, Ataturk University, Erzurum 25240, Turkey; 9 Department of Cardiac Surgery, University General Hospital of Heraklion, Medical School, University of Crete, 71003 Heraklion, Greece; 10 Department of Forensic Sciences and Toxicology, Faculty of Medicine, University of Crete, 71003 Heraklion, Greece; 11 Department of Cardiology, University General Hospital of Larissa, 41110 Larissa, Greece

**Keywords:** DEN, artichoke, oxidative stress, apoptosis, Klotho, PPARγ

## Abstract

(1) Background: Various epidemiological studies suggest that oxidative stress and disrupted neuronal function are mechanistically linked to neurodegenerative diseases (NDs), including Parkinson’s disease (PD) and Alzheimer’s disease (AD). DNA damage, oxidative stress, lipid peroxidation, and eventually, cell death such as NDs can be induced by nitrosamine-related compounds, leading to neurodegeneration. A limited number of studies have reported that exposure to diethylnitrosamine (DEN), which is commonly found in processed/preserved foods, causes biochemical abnormalities in the brain. Artichoke leaves have been used in traditional medicine as a beneficial source of bioactive components such as hydroxycinnamic acids, cynarine, chlorogenic acid, and flavonoids (luteolin and apigenin). The aim of this study is to investigate the favorable effects of exogenous artichoke (Cynara scolymus) methanolic leaf extract supplementation in ameliorating DEN-induced deleterious effects in BALB/c mouse brains. (2) Methods: This study was designed to evaluate DEN (toxicity induction by 100 mg/kg) and artichoke (protective effects of 0.8 and 1.6 g/kg treatment) for 14 days. All groups underwent a locomotor activity test to evaluate motor activity. In brain tissue, oxidative stress indicators (TAC, TOS, and MDA), Klotho and PPARγ levels, and apoptotic markers (Bax, Bcl-2, and caspase-3) were measured. Brain slices were also examined histopathologically. (3) Results: Artichoke effectively ameliorated DEN-induced toxicity with increasing artichoke dose. Impaired motor function and elevated oxidative stress markers (decreasing MDA and TOS levels and increasing TAC level) induced by DEN intoxication were markedly restored by high-dose artichoke treatment. Artichoke significantly improved the levels of Klotho and PPARγ, which are neuroprotective factors, in mouse brain tissue exposed to DEN. In addition, caspase-3 and Bax levels were reduced, whereas the Bcl-2 level was elevated with artichoke treatment. Furthermore, recovery was confirmed by histopathological analysis. (4) Conclusions: Artichoke exerted neuroprotective effects against DEN-induced brain toxicity by mitigating oxidant parameters and exerting antioxidant and antiapoptotic effects. Further research is needed to fully identify the favorable impact of artichoke supplementation on all aspects of DEN brain intoxication.

## 1. Introduction

Oxidative stress (OS) is considered the sum of all biochemical processes, leading to an imbalance between oxidants and antioxidants in living organisms [1]. This imbalance occurs as a consequence of an excess generation of reactive oxygen species (ROS) or of inadequate antioxidant system functioning [2]. Research conducted over the past few decades has brought to light the widespread involvement of OS in many neurodegenerative diseases (NDs), including Parkinson’s disease (PD), Alzheimer’s disease (AD), and amyotrophic lateral sclerosis (ALS) [2,3]. Nitrosamines, which are widely used as preservatives in processed/preserved foods and drug preparations, lead to neurodegeneration with several molecular and biochemical features of NDs [4,5]. With the current increasing incidence of NDs, it is necessary to identify and understand the mechanisms by which nitrosamines affect the brain and increased the risk of NDs.

In this regard, we aimed to investigate the effects of diethylnitrosamine (DEN) on the male mouse brain in terms of OS. DEN is one of the chemical carcinogens to which people are highly exposed in their daily lives [6]. Water and food items including cured and grilled meats, tobacco products and pharmaceutical preparations are potent sources of human exposure to DEN [6,7]. This exposure leads to toxic and mutagenic sequelae occurring as a result of increased production of ROS such as superoxide and hydrogen peroxide, leading to elevated OS, lipid peroxidation, and DNA damage [4,8,9]. OS and DNA damage activate the apoptotic pathway, which results in cell death [10]. 

Recently, various proteins and signaling pathways involved in the OS mechanism have been proposed. Klotho protein is predominantly expressed in the brain and protects neurons from oxidative damage [11], exhibiting neuroprotective potential, mainly by activating the antioxidant enzymatic system, decreasing the levels of oxidized lipids and DNA, and reducing the number of apoptotic cells in NDs [12]. On the other hand, Klotho expression can be modified by peroxisome proliferation-activated receptor γ (PPARγ) [13]. PPARγ downregulation can reduce the antioxidative and antiapoptotic protective mechanisms in the brain and could be responsible for the degenerative process related to NDs [14,15]. Through their diverse functions in the brain, Klotho/PPARγ signaling has become a novel therapeutic target for NDs such as Alzheimer’s disease [13,16,17]. Therefore, the discovery of Klotho/PPARγ enhancers may enable novel treatment options for DEN-related brain disorders.

Artichoke, *Cynara scolymus* L., is a member of the *Asteraceae* family, and its leaves have been used in traditional medicine for many years to treat various health problems [18]. It is a beneficial source of bioactive compounds such as hydroxycinnamic acids, cynarine, chlorogenic acid, and flavonoids (luteolin, and apigenin) [18,19]. Phenolic components found in artichoke exhibit scavenging activity against ROS and free radicals and act as a protective shield against oxidative damage to biological molecules, such as proteins, lipids, and DNA [19,20]. Additionally, a recent study reported that artichokes have beneficial effects on the brain through the regulation of lipid metabolism and the alleviation of OS [21]. Artichoke has been known an indispensable food for a balanced diet for humans for centuries, owing to its many health benefits. The artichoke is native to the Mediterranean region. The artichoke industry is currently largely based in California. Fresh artichoke is most commonly found between the months of February and June or September and December, when the plant is in season. It seems that Egyptians and Mesopotamian people knew of this plant and used it, especially for its effects on the digestive apparatus [22]. Artichoke was a luxury product for Greeks and Romans, owing to its aphrodisiac properties, and remained a delicacy for the elite until the 18th century. At the beginning of the 20th century, artichoke increased in popularity. Artichoke is recommended for pregnancy, especially during the third trimester, owing to its richness in microminerals. Recently, the market has focused of producing artichoke concentrates as food supplements, which are available as pills, ampules, capsules, powders, teas, and other formulations. The recommended daily dose for an adult is three servings of between 500 and 650 milligrams of artichoke leaf standardized extract. The intake should last between a month and a month and a half [23].

Although the protective effects of artichoke against tissue damage have been demonstrated in some studies [19,21], its effects on DEN intoxication-induced brain injury have not been studied. The aim of this study is the detailed description of the mechanisms of DEN-induced neurotoxicity and the evaluation of the protective effects of artichoke administration on DEN-induced oxidative brain damage in mice by employing behavioral, biochemical, and histopathological study methods.

## 2. Materials and Methods

### 2.1. Chemical and Reagents

Thiopental sodium was obtained from IE Ulagay-Turkey. We purchased DEN (Cas Number # 55-18-5) and phosphate buffer sodium (PBS) from Sigma-Aldrich (Oakville, ON, Canada; St. Louis, MO, USA, Cas Number # 7558-80-7). A Thermo Nalgene™ Rapid-Flow™ filter was obtained from Thermo Fisher Waltham (Canada; Waltham, MA, USA, Cas Number # 64-19-7). A Sephadex LH20 column was purchased from Merck (Darmstadt, Germany, Cas Number # 9041-37-6). Hematoxylin and eosin (H&E) were obtained from Merck (Darmstadt, Germany, Cas Number # 17372-87-1). Malondialdehyde (MDA) (Cat. No. E-BC-K025-M), Klotho (Cat. No. E-EL-M3051), PPAR-gamma (PPARγ) (Cat. No. E-EL-M0893), Bax (Cat. No. E-EL-M0178), Bcl-2 (Cat. No. E-EL-M0175), and caspase-3 (Cat. No. E-EL-M0238) were obtained from (Elabscience, Houston, TX, USA).

### 2.2. Methanolic Extraction of Artichoke Leaves

The material studied was C. scolymus L. cv. Sakiz grown in Aydin province during the period of May 2020–2021. Artichoke leaves were washed 5 times with distilled water to remove impurities and then dried at 60 °C in an air-drying oven. The dried materials were ground to a powder in a laboratory blender. Then, 200 g of powdered artichoke leaves was shaken in a flask with 1 L of methanol at 150 RPM at 4 °C for 24 h in an incubator. Afterward, the obtained extract was filtered under vacuum with a Thermo Nalgene™ Rapid-Flow™ filter with a pore diameter of 0.45 µm. The methanol in the obtained extract was concentrated in a rotary evaporator at 40 °C. The concentrated extract was dissolved in methanol and passed through a Sephadex LH20 column, and the obtained eluates were completely dried in a rotary evaporator at 40 °C [24]. 

### 2.3. Animals and Experimental Protocol

A total of 32 male BALB/c (18–20 g) [25] mice were initially maintained under normal conditions (24 ± 1°C with a 12:12 h reverse lighting cycle) with free access to chow and water. The BALB/c mice were randomly divided into 4 groups of 8 mice each group: control group (C), DEN control group (DEN), DEN + 0.8 g/kg artichoke-treated group (DEN  +  A1), and DEN + 1.6 g/kg artichoke-treated group (DEN  +  A2) [26,27]. Animals were administered i.p with 100 mg/kg DEN for three days in the first week. Artichoke was dissolved in 0.9% saline and administered by gavage daily for 14 days [28,29,30]. The control group was treated with a similar volume of saline to eliminate the effects of the vehicle. 

### 2.4. Assessment of Locomotor Function

Locomotion was evaluated with a photoelectric apparatus (MAY 9908, Comment, Ankara, Turkey), which involved a plexiglass cage (42 cm × 42 cm × 30 cm). With the photobeam deductions, the subsequent movements were recorded for 10 min: horizontal and ambulatory activity, total distance, and resting time [31].

### 2.5. Sample Preparation

All mice were sacrificed with a high dose of (50 mg/kg) sodium-thiopental anesthesia. Brains were then dissected and homogenized with PBS, and homogenates were centrifuged (3000× *g*, 7 min, 4 °C). Supernatants were stored at −86 °C for biochemical analysis until use. 

### 2.6. Measurement of Oxidative Stress

Biochemical analyses of total antioxidant capacity (TAC) (Cat no: OK20115A) [32] and total oxidant status (TOS) (Cat no: OK201270) [33] were performed accordingly to the manufacturer’s instructions (Rel Assay Diagnostics, Gaziantep, Turkey). Lipid peroxidation was determined by measuring MDA levels with commercially available kits accordingly to the manufacturer’s directions.

### 2.7. Measurement of Klotho and PPARγ Protein Levels 

Levels of Klotho and PPARγ protein were measured in brain homogenates using a mouse ELISA kit according to the manufacturer’s directions. Briefly, the samples were added to wells and incubated for 90 min at 37 °C. Biotinylated detection Ab working solutions were added and incubated for 60 min. Then, the plate was washed, and horseradish peroxidase (HRP) conjugate working solution was added and incubated for 30 min at 37 °C. Finally, substrate reagent and stop solution were added, and the plate was read at 570 nm using a Multiskan™ GO microplate spectrophotometer reader (Thermo Scientific, Janakkala, Finland).

### 2.8. Measurement of Apoptotic Marker Levels

Levels of Bax, Bcl-2, and caspase-3 protein were determined in brain homogenates using a mouse ELISA kit according to the manufacturer’s instructions. First, samples were added to wells at the appropriate volume and incubated at 37 °C for 90 min. After discarding the liquid, biotinylated detection Ab working solution was added to each well and incubated under appropriate conditions. HRP conjugate was used for the working solution. The stop solution was added to the substrate solution after 15 min of incubation. Finally, the plate was read at 570 nm with a Multiskan™ GO Microplate spectrophotometer reader (Thermo Scientific, Janakkala, Finland).

### 2.9. Routine Histological and Hematoxylin Eosin (H&E) Procedure

Histopathological analysis was conducted as previously described. Briefly, brain tissues taken for histopathological evaluation were fixed in 10% formalin solution for 48 h. The tissues were then processed through routine procedures including dehydration and removal of fixatives and then fixed in paraffin blocks. The sections were stained with H&E, examined, and photographed under a light microscope (Olympus BX51, Tokyo, Japan) [34]

### 2.10. Statistical Analysis

All data were presented as means plus the standard deviation (mean ± SD). Behavioral and ELISA findings were analyzed with one-way ANOVA followed by Tukey’s post hoc test. Histopathological findings were analyzed by the non-parametric Kruskal–Wallis test with a Mann–Whitney post hoc U test. *p*-values < 0.05 were considered significant.

## 3. Results

### 3.1. Locomotor Activity Results

**Figure 1:**
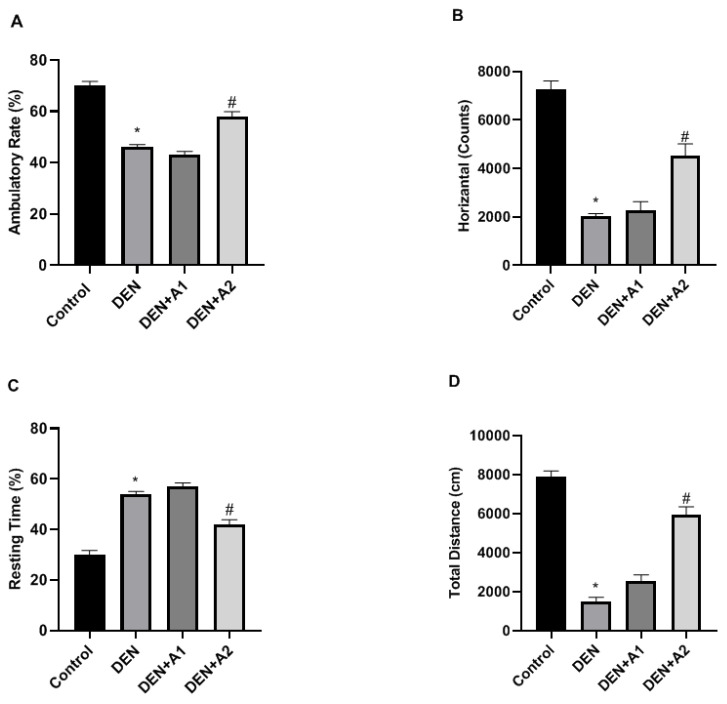
The effects of artichoke on (**A**) Ambulatory rates (%), (**B**) Horizontal activities (Counts), (**C**) Resting time (%), and (**D**) Total distance (cm) in DEN-intoxicated mice (*n* = 8). Data are presented as means  ±  SD, * *p* < 0.05, # *p* < 0.05 vs. DEN group; DEN: diethylnitrosamine, A1: 0.8 g/kg Artichoke, A2: 1.6 g/kg artichoke.

Ambulatory activity was significantly reduced in the DEN group (46 ± 1.09%, *p* < 0.05) compared to the control group (70 ± 1.73%). However, there was a marked elevation in ambulatory activity in the treatment groups, especially in the DEN + A2 group (58 ± 1.87%, *p* < 0.05) in comparison to the DEN group (Figure 1A). In the control mice, horizontal movement was measured at 7247 ± 360.87, whereas it was significantly diminished in DEN-treated mice (2012 ± 120.44, *p* < 0.05). The DEN-induced reduction in horizontal movements was restored in the DEN + A2 group (4529 ± 480.18, *p* < 0.05) (Figure 1B).

On the other hand, in control mice, the total resting time was 30 ± 1.73%, which increased significantly in DEN-treated mice (54 ±1.41%, *p* < 0.05). The DEN-induced increases in total resting time were reduced in mice treated with a high dose of artichoke (42 ± 1.87%, *p* < 0.05) (Figure 1C). However, there was no change in total resting time in the DEN + A1 group compared to the DEN group. Compared to the controls (7889 ± 300.87 cm), mice with DEN intoxication covered a significantly reduced total distance (1501 ± 200.44 cm, *p* < 0.05). The DEN-induced reduction in total distance was restored partially but not significantly by A1 treatment (2550 ± 300.16 cm), whereas, it recovered completely in mice in the A2 treatment group (5949 ± 400.18 cm, *p* < 0.05), (Figure 1D). The decrease in total distance resulting from DEN-induced toxicity improves with artichoke supplementation.

### 3.2. Results of Oxidative Stress

**Figure 2:**
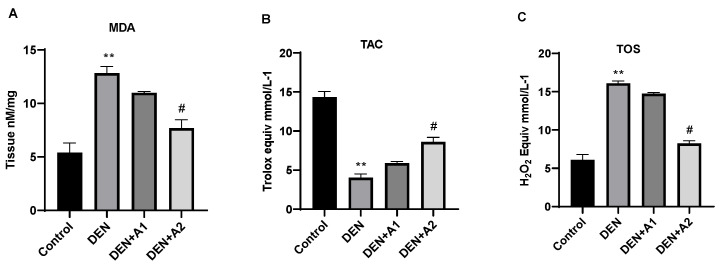
Effects of artichoke on (**A**) Malondialdehyde (MDA) levels (nM/mg), (**B**) Total Antioxidant Capacity (TAC) (Trolox Equiv mmol/L^−1^), and (**C**) Total Oxidative Status (TOS) (H_2_O_2_ mmol Equiv/L^−1^) levels in brain tissue (*n* = 8). Data are presented as means  ±  SD, ** *p* < 0.001 vs. control group, # *p* < 0.05 vs. DEN group; DEN: diethylnitrosamine, A1: 0.8 g/kg artichoke, A2: 1.6 g/kg artichoke.

As shown in Figure 2A, DEN administration significantly (*p* < 0.001) elevated MDA (12.83 ± 0.61 nM/mg) levels compared with the normal control group (5.39 ± 0.89 nM/mg). In contrast, in the DEN + A2 group, a significant decrease in MDA levels was observed compared to the DEN group. Additionally, MDA levels in the high-dose artichoke group (7.68 ± 0.81; *p* < 0.05) approached those of the control group. Compared with the control animals (14.36 ± 0.70 Trolox Equiv mmol/L^−1^), DEN decreased TAC levels significantly (4.02 ± 0.49 Trolox Equiv mmol/L^−1^; *p* < 0.001). The DEN-induced decrease in TAC levels was prevented by the high-concentration artichoke treatment (8.607 ± 0.57 Trolox Equiv mmol/L^−1^; *p* < 0.05) (Figure 2B). DEN administration significantly increased TOS levels (16.09 ± 0.31 H_2_O_2_ mmol Equiv/L^−1^, *p* < 0.001) compared to the control group (6.084 ± 0.7 H_2_O_2_ mmol Equiv/L^−1^) (Figure 2C). However, a significant reduction in TOS levels was observed in the DEN + A2 group (8.24 H_2_O_2_ mmol Equiv/L^−1^, *p* < 0.05) compared to the DEN group. 

### 3.3. Results of PPARγ and Klotho Levels

**Figure 3:**
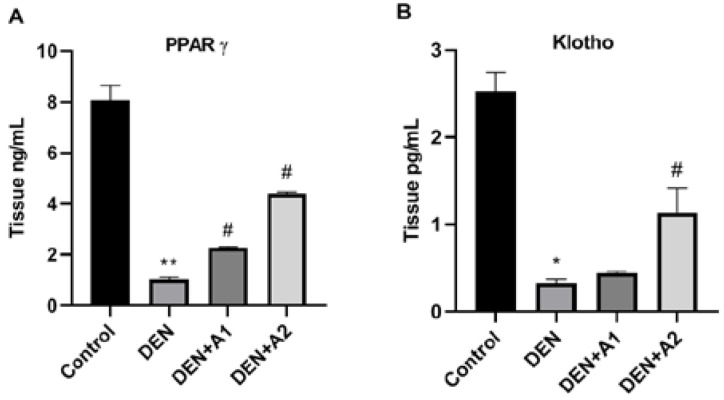
Effects of artichoke on (**A**) PPARγ (Tissue ng/mL) and (**B**) Klotho (Tissue ng/mL) levels in brain tissue (*n* = 8). Data are presented as means ±  SD * *p* < 0.05, ** *p* < 0.001 vs. control group, # *p* < 0.05 vs. DEN group; DEN: diethylnitrosamine, A1: 0.8 g/kg artichoke, A2: 1.6 g/kg artichoke.

Regarding brain tissue PPARγ protein levels, very high levels were found in the control group (Figure 3A). Compared with control mice (8.06 ± 0.59 ng/mL), PPARγ protein levels were significantly decreased in mice that received DEN (1.025 ± 0.08 ng/mL, *p* < 0.001). The high-dose artichoke treatment prevented a DEN-induced reduction in PPARγ levels (4.390 ± 0.45 ng/mL). PPARγ protein levels in the DEN + A2 group were significantly increased (*p* < 0.05) by almost 4.5 times compared to the DEN group. However, no difference was observed in PPARγ levels between the DEN + A1 and DEN groups. Similarly to PPARγ, Klotho brain levels were found to be increased in control mice. In the DEN group, a significant reduction in Klotho levels (0.3282 ± 0.42 pg/mL; *p* < 0.05) was observed compared to the control group (2.548 ± 0.22 pg/mL). Although no significant change in Klotho levels was observed in the DEN + A1 group compared to the DEN group, a significant increase in Klotho levels (1.13 ± 0.28 pg/mL) was observed in the DEN + A2 group (Figure 3A).

### 3.4. Results of Apoptotic Protein Levels

**Figure 4:**
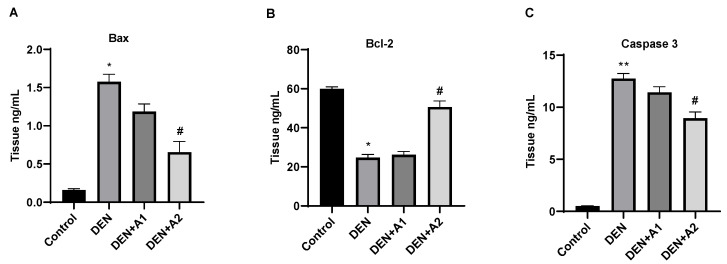
Effects of artichoke on (**A**) Bax (Tissue ng/mL), (**B**) Bcl-2 (Tissue ng/mL), and (**C**) caspase-3 (Tissue ng/mL) levels in brain tissue (*n* = 8). Data are presented as means  ±  SD * *p* < 0.05, ** *p* < 0.001 vs. control group, # *p* < 0.05 vs. DEN group; DEN: diethylnitrosamine, A1: 0.8 g/kg artichoke, A2: 1.6 g/kg artichoke.

Bax brain tissue levels were markedly elevated in response to DEN administration compared to control mice (1.57 ± 0.084 ng/mL and 0.16 ± 0.016, respectively; *p* < 0.05). Low-dose artichoke treatment was insufficient to eliminate brain apoptotic damage (1.186 ± 0.097 ng/mL). Furthermore, high-dose artichoke treatment protected cells from apoptotic damage and decreased Bax (0.65 ± 0.14 ng/mL; *p* < 0.05) levels compared to the DEN group (Figure 4A). Regarding Bcl-2 levels, DEN administration significantly reduced Bcl-2 levels (*p* < 0.05) compared to the control group. However, the DEN-induced reduction in Bcl-2 levels was restored following high-dose artichoke administration (50.60 ± 3.15 ng/mL; *p* < 0.05) (Figure 4B). No increase in caspase-3 levels was observed in the control group. DEN administration resulted in a prominent elevation in caspase-3 (11.43 ± 0.53 ng/mL; *p* < 0.001) levels compared to the control group. However high-dose artichoke treatment attenuated caspase-3 levels (8.93 ± 0.60 ng/mL, *p* < 0.05) compared to the DEN-treated group (Figure 4C).

### 3.5. Histopathological Results

**Figure 5:**
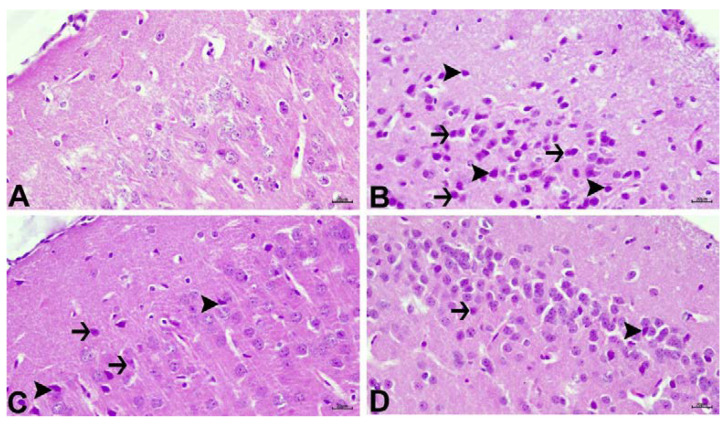
(**A**) Control group with normal histological appearance. (**B**) DEN group with intense degeneration (arrows) and necrosis (arrowheads) of neurons. (**C**) DEN + A1 with moderate degeneration (arrows) and necrosis (arrowheads) in neurons. (**D**) DEN+ A2 with mild degeneration (arrows) and necrosis (arrowheads) in neurons. DEN: diethylnitrosamine, A1: 0.8 g/kg artichoke, A2: 1.6 g/kg artichoke; H&E, Bar: 20 µm.

The findings of the histopathological examination are shown in Figure 5. In the control group, a normal histological appearance was observed (Figure 5A). Severe degeneration and necrosis in neurons and intense hyperemia in meningeal and parenchymal vessels were observed in the DEN group (Figure 5B). Moderate degeneration and necrosis in neurons, as well as strong hyperemia in arteries, was detected in the DEN + A1 group (Figure 5C). The DEN + A2 group, on the other hand, showed mild vertical degeneration and necrosis in neurons, as well as moderate hyperemia in arteries (Figure 5D). Histopathological findings are summarized in Table 1. 

## 4. Discussion

Previous studies reported that exposure to preservatives and chemicals in food and drug preparations can lead to numerous NDs such as Alzheimer’s disease [4,5]. OS is a commonly cited mechanistic feature in NDs [2,3]. Abnormal accumulation of ROS, such as superoxide anions and hydroxyl radicals, damage macromolecules, such as DNA, RNA, lipids, and proteins, leading to neurodegeneration [2]. Furthermore, reports have demonstrated that oxidative stress participates in the progression of motor disturbance in animal models of NDs [35,36]. In this study, the effect of artichoke on DEN-induced brain injury in mice was examined behaviorally, biochemically, and histopathologically. In the mice administered DEN, the impairment of locomotor activity was manifested by increased rest time accompanied by a reduction in total distance and ambulatory and horizontal movement activities. Consistent with the current findings, impairment in locomotor functions caused by DEN toxicity was presented in a report by Isobe et al. [37]. High-dose artichoke treatment alleviated the deleterious brain effects of DEN, as manifested by improved locomotor activity, in accordance with previous reports [21]. 

Experimental and epidemiological research has provided evidence connecting nitrosamine exposure via food to the pathogenesis of primary brain tumors, suggesting that these compounds are cleaved into the brain and lead to disease [4,8]. It has been reported that oxidative metabolism, mitochondrial function, ATP production, and cell survival are impaired as a result of radical ion accumulation [38]. Suzanne et al. suggested that nitrosamines induce neurodegeneration with several pathological features resembling Alzheimer’s, including elevated levels of oxidative damage, DNA injury, and Tau immunoreactivity [4]. On the other hand, DEN-induced neurodegeneration is related to an increase in ROS, membrane lipid peroxidation, and a reduction in antioxidants [4,5,8]. In this research, OS with disturbed redox balance was found in DEN-treated mouse brains, as manifested by a marked increase in MDA levels, a product of lipid peroxidation, and by elevated TOS levels associated with a marked decrease in TAC levels—findings that are in line with those of previous studies. However, high-dose artichoke treatment significantly suppressed the elevation of MDA and TOS concentrations and strengthened the antioxidant defense mechanism by increasing TAC levels. Ibrahim et al. previously showed the neuroprotective effects of artichoke in reducing lipid peroxidation and elevating the antioxidant enzyme synthesis [21]. 

To further elucidate the mechanism of OS caused by DEN in the brain, we investigated the Klotho/PPARγ signaling pathway. It has been reported that the reduction in Klotho levels related to excessive ROS generation is closely linked to the causal mechanism of NDs [39,40]. In the current research, we found that the reduction in Klotho protein levels occurred in association with oxidative damage in DEN-intoxicated mouse brains. There have been no reports in the literature regarding Klotho activity in DEN-induced brain damage. Kuang et al. demonstrated that Klotho upregulation provides resistance to OS by strengthening the intrinsic mechanism of the antioxidant system [11]. Another study reported that Klotho-deficient mice exhibit elevated levels of oxidized lipids and DNA in the hippocampus [40]. However, the underlying mechanisms of Klotho reduction have not yet been fully clarified [13]. Therefore, we tested the role of the target gene, PPARγ, in the regulation of Klotho expression after DEN administration. In this study, DEN-induced brain toxicity was associated with a decrease in PPARγ protein levels concomitant with a reduction in Klotho levels. Consistent with the current findings, Zhang showed that Klotho levels can be affected by PPARγ activation in a mouse model of traumatic brain injury [13]. However, high-dose artichoke treatment ameliorated the degenerating effects on mice brains and increased Klotho and PPARγ concentrations in harmony with decreased lipid peroxidation and elevated total antioxidant levels. Although no studies have been published regarding the relationship between artichoke and Klotho and PPARγ activity, recent studies demonstrated that phenolic compounds have neuroprotective effects in brain injuries by upregulating Klotho and PPARγ expression [39,41]. It was also reported that the regulation of Klotho levels via PPARγ signaling contributes to the protection of rat brains in ischemic preconditioning [13]. 

Neuronal apoptosis induced by OS is a significant pathological manifestation in NDs [42]. Increased membrane permeability as a result of Bax activation induces apoptosis related to mitochondrial dysfunction. Furthermore, Bax activation inhibits Bcl-2, which has a repressive effect on apoptosis and ultimately promotes apoptosis [43]. It has also been established that caspase-3 is a key enzyme in triggering apoptosis [44]. In the present study, Bax and caspase-3 levels were elevated, whereas Bcl-2 levels were reduced in DEN-intoxicated mice. This finding, consistent with previous reports, suggests the involvement of apoptosis in the neuronal death in NDs [45,46]. Artichoke apoptosis suppression and the importance of apoptosis in the formation of NDS led to the investigation of the favorable effects of artichoke in DEN-induced brain toxicity. In the current study, artichoke treatment counteracted apoptosis by reducing caspase-3 and Bax levels and elevating Bcl-2 levels, suggesting that artichoke attenuates apoptosis with its antioxidant and antiapoptotic properties.

These findings were supported by the histopathological analysis of DEN-exposed mice, which presented with necrosis and severe hyperemia in meningeal and parenchymal vessels, in line with a report by Tong at al. [47]. High-dose artichoke treatment ameliorated neuronal cells histopathological findings. The results of the current study are in agreement with earlier reports showing that artichoke has a neuronal protective effect, owing to its antioxidant properties [21]. 

## 5. Conclusions

In summary, the present study shows, for the first time, an association between Klotho/PPARγ signaling related to oxidative damage and apoptosis in DEN-induced neurotoxicity. Moreover, high-dose artichoke administration prevented the development of neuropathologies and motor disturbance in DEN-intoxicated mice. The underlying mechanism of the reported favorable effect likely involves an increase in Klotho/PPARγ and the concomitant amelioration of brain oxidative damage and apoptosis. 

## Figures and Tables

**Table 1:** H&E staining scores of brain tissues.

	C Group	DEN Group	DEN + A1 Group	DEN + A2 Group
**Degeneration in neurons**	−	+++	++	+
**Necrosis in neurons**	−	+++	++	+
**Hyperemia**	−	+++	+++	++

Grade 0: − (0% negative), Grade 1: + (0–33% mild positive), Grade 2: ++ (33–66% moderate positive), and Grade 3: +++ (66–100% severe positive). HE: hematoxylin and eosin staining, C: control, DEN: diethylnitrosamine, A1: 0.8 g/kg artichoke, A2: 1.6 g/kg artichoke.

## Data Availability

The datasets used and/or analyzed during the current study are available from Bilecik Seyh Edebali University, Faculty of Medicine, Department of Pharmacology, upon reasonable and justifiable request according to the rules and procedures of the University.
